# OSBP-Related Protein 8 (ORP8) Regulates Plasma and Liver Tissue Lipid Levels and Interacts with the Nucleoporin Nup62

**DOI:** 10.1371/journal.pone.0021078

**Published:** 2011-06-15

**Authors:** Tianhong Zhou, Shiqian Li, Wenbin Zhong, Terhi Vihervaara, Olivier Béaslas, Julia Perttilä, Wei Luo, Yingliang Jiang, Markku Lehto, Vesa M. Olkkonen, Daoguang Yan

**Affiliations:** 1 Department of Biology, Jinan University, Guangzhou, China; 2 Minerva Foundation Institute for Medical Research, Biomedicum, Helsinki, Finland; 3 Folkhälsan Institute of Genetics, Folkhälsan Research Centre Biomedicum, Helsinki, Finland; 4 Institute of Biomedicine/Anatomy, University of Helsinki, Helsinki, Finland; University of Geneva, Switzerland

## Abstract

We earlier identified OSBP-related protein 8 (ORP8) as an endoplasmic reticulum oxysterol-binding protein implicated in cellular lipid homeostasis. We now investigated its action in hepatic cells *in vivo* and *in vitro*. Adenoviral overexpression of ORP8 in mouse liver induced a decrease of cholesterol, phospholipids, and triglycerides in serum (−34%, −26%, −37%, respectively) and liver tissue (−40%, −12%, −24%), coinciding with reduction of nuclear (n)SREBP-1 and -2 and mRNA levels of their target genes. Consistently, excess ORP8 reduced nSREBPs in HuH7 cells, and ORP8 overexpression or silencing by RNA interference moderately suppressed or induced the expression of SREBP-1 and SREBP-2 target genes, respectively. In accordance, cholesterol biosynthesis was reduced by ORP8 overexpression and enhanced by ORP8 silencing in [^3^H]acetate pulse-labeling experiments. ORP8, previously shown to bind 25-hydroxycholesterol, was now shown to bind also cholesterol *in vitro*. Yeast two-hybrid, bimolecular fluorescence complementation (BiFC), and co-immunoprecipitation analyses revealed the nuclear pore component Nup62 as an interaction partner of ORP8. Co-localization of ORP8 and Nup62 at the nuclear envelope was demonstrated by BiFC and confocal immunofluorescence microscopy. Furthermore, the impact of overexpressed ORP8 on nSREBPs and their target mRNAs was inhibited in cells depleted of Nup62. Our results reveal that ORP8 has the capacity to modulate lipid homeostasis and SREBP activity, probably through an indirect mechanism, and provide clues of an entirely new mode of ORP action.

## Introduction

Cellular cholesterol biosynthesis and uptake, as well as fatty acid biosynthesis, are controlled by transcription factors named sterol regulatory element binding proteins (SREBPs) and their sterol-sensing accessory factor, the SREBP cleavage activating protein (SCAP) [1]–[3]. The SREBPs are synthesized as precursors anchored to endoplasmic reticulum (ER) membranes and complexed with SCAP. When the cellular cholesterol level is low, SREBP-SCAP complexes move to the Golgi apparatus, where SREBPs undergo a two-step proteolytic processing, leading to the release of an N-terminal fragment, basic helix-loop-helix leucine zipper transcription factor. These factors enter the nucleus where they bind to sterol regulatory elements (SRE) in the promoter regions of a number of genes whose products mediate the synthesis of cholesterol and fatty acids.

Bidirectional nucleocytoplasmic transport occurs through nuclear pore complexes (NPC) embedded in the nuclear envelope. The vertebrate NPC is a 60–125 MDa structure composed of approximately 30 distinct proteins called nucleoporins [4], [5]. Large macromolecules are actively transported through its central channel, while small molecules may diffuse through one of the eight smaller channels in the symmetrical spoke-ring complex. Large proteins carrying a classical nuclear localization signal (NLS) are transported into the nucleus through the following multistep process: The NLS is recognized by the adaptor protein importin-α (karyopherin-α). This complex travels to the periphery of the nucleus where it is bound by a NPC-associated protein, importin-β, and the entire complex is transported through the NPC into the nucleus. A subset of the nucleoporins, including Nup62, are characterized by phenylalanine-glycine rich (FG) repeat sequences and fill the central channel of the NPC. The FG domains have an unfolded structure and are responsible for interaction with importin-cargo complexes moving through the pore [6], [7].

Oxysterol binding protein (OSBP) is a cytoplasmic protein with affinity for several oxysterols [8]–[10]. It plays a role in the trafficking of ceramide from the ER to the Golgi apparatus for sphinghomyelin synthesis [11], and acts as a sterol-dependent scaffold that regulates the activity of extracellular signal-regulated kinases, ERK [12]. Families of proteins displaying sequence homology to the carboxyl terminal sterol-binding domain of OSBP are present in most eukaryotic organisms [13]–[15]. In humans the gene/protein family consists of 12 members [16], [17]. The mammalian OSBP-related proteins (ORPs) have been implicated as sterol sensors that regulate cellular functions ranging from sterol and neutral lipid metabolism to vesicle transport and cell signaling [18].

ORP8 is a member of the ORP family, with a trans-membrane segment at its C-terminus specifying localization at the ER. We have previously reported that ORP8 impacts the expression of ABCA1 and cellular cholesterol efflux [19]. We now report functional characterization of ORP8 in hepatic cells and its interaction with the nucleoporin Nup62, and provide evidence that ORP8 regulates the abundancy of active nuclear SREBPs thus impacting lipid metabolism *in vivo* and *in vitro*.

## Methods

### Antibodies and other reagents

Rabbit antibodies against human ORP8 were produced and affinity-purified as described [19]. Mouse monoclonal anti-SREBP-2 (clone 1C6) was from BD Biosciences (San Jose, CA), anti-SREBP-1 and anti-Nup62 from ProteinTech (Chicago, IL), anti-SREBP-1 from Abcam (Cambridge, UK), and monoclonal anti-actin (clone JLA20) from the Developmental Studies Hybridoma Bank (Univ. of Iowa).

### cDNA constructs and transfection

Full-length human *ORP8* cDNA (accession number NM_001003712) was inserted into the XbaI site of pcDNA4HisMaxC (Invitrogen, Carlsbad, CA). Transient transfections of cultured cells were carried out using Lipofectamine 2000 (Invitrogen) according to the manufacturer's instructions, or by using the Neon™ electrotransfection System (Invitrogen), achieving >90% transfection frequency. Human ORP1L (AF323726), ORP3 (NM_015550), and ORP10 (NM_017784) cDNAs in pcDNA4HisMaxC were used for immunofluoresecence microscopy studies. For recombinant protein production, the ORP8 ORD (aa 242–828) was subcloned into pHAT2 (Dr. Johan Peränen, Institute of Biotechnology, Univ. of Helsinki).

### Construction of recombinant adenoviruses

The human ORP8 cDNA was inserted into pAdenovator-CMV5-IRES-GFP (QbioGene, Illkirch, France), and recombinant adenoviruses (AdORP8) were generated in HEK293 cells using the AdEasy system according to the manufacturer's instructions. The viruses constructed with this vector encode GFP under an IRES sequence. A control adenovirus encoding GFP alone (AdGFP) was generated from the plain pAdenovator transfer vector. The recombinant viruses were plaque purified, expanded, and purified with kit no:631533 from Clontech (Mountain View, CA).

### Intravenous injection of C57B/6 mice

Female C57B/6JOlaHsd mice were purchased from the animal center of Guangdong Province (Guangzhou, China), housed in a humidity (40–50%) and temperature (21–22°C) controlled room with a 12∶12 h dark/light cycle, and maintained on Rat&Mouse Maintenance Diet. For adenovirus injections, 10-week-old animals were calmed with Hypnorm-Dormicum and injected through the tail vein with 3×10^8^ pfu of adenovirus in 100 l PBS. At 5 days after injection, the animals were fasted for 6 h, and blood and liver tissue samples were collected. One mg/ml EDTA was immediately added in the blood samples and plasma prepared by centrifugation. All experimental procedures involving animals were performed in accordance with the Guide for the Care and Use of Laboratory Animals (NIH publications Nos. 80–23, revised 1996) and according to the institutional ethical guidelines for animal experiments. The experimental protocols were approved by the Ethics Committee for Animal Experiments of Jinan University (permit number 2009210).

### Analysis of plasma and liver tissue lipids

Total plasma cholesterol (Cat. no k603, Biovision, Mountain View, CA), choline-containing phospholipids (Cat. no pl9220, Jinhao, Beijing, China) and triglycerides (Cat. no etga-200, Bioassay Systems, Haywards, CA) were measured using fully enzymatic methods. The total cholesterol assay protocol includes hydrolysis of cholesterol esters by cholesterol esterase provided with the kit. The phospholipid analysis involves phospholipase D digestion to release choline which is quantified by a color reaction after choline oxidase and peroxidase steps [20]. The Bioassay Systems triglyceride assay is based on triglyceride hydrolysis by triacylglycerol acylhydrolase and quantification of the glycerol released in one step [21].

Sections of liver tissue were excised, snap-frozen in liquid nitrogen and stored at the temperature of −70°C. Lipids were extracted from the tissue: Briefly, liver tissue (approximately 100 mg) was homogenized and sonicated in 1 ml of 95% methanol and mixed with 2 ml of chloroform. The organic phase was washed with 0.9% NaCl solution and dried under nitrogen. The residuals were dissolved in 200 µl of tetraethylammoniumhydroxide (diluted 1∶28 with 95% ethanol) and incubated at 60°C for 30 min with 200 µl of 0.05 M HCl. The formed glycerol was measured enzymatically (Kit 1488872, Roche Diagnostics, Basel, Switzerland). Total cholesterol and choline-containing phospholipids (PC, lyso-PC, SM) were measured for the same tissue specimens from the solvent phase after initial chloroform-methanol extraction, using the assays specified above for serum samples.

### Cell culture

The human hepatoma cell line HuH7 [22] was cultured in Eagle's minimal essential medium with Earle's salts (EMEM, Sigma-Aldrich, St. Louis, MO), 20 mM Hepes, pH 7.4, 10% foetal bovine serum (FBS), penicillin (100 I.U./ml) and streptomycin (100 g/ml). The cells were maintained in 5% CO_2_, 37°C.

### Analysis of nuclear SREBP

Nuclear fractions of liver tissue infected by adenoviruses and HuH7 cells transfected with *ORP8* cDNA or *Nup62* siRNA were isolated according to Blanquart et al. [23]. The specimens were Western blotted using antibodies against SREBP-1 and -2.

### Quantitative real-time RT-PCR

Total RNA was isolated with RNeasy Mini kit (Qiagen, Valencia, CA), reverse-transcribed using Superscript II (Invitrogen) and each RNA sample was amplified in triplicate for the genes of interest and β-actin as a housekeeping marker, on a 7000 or 7400 Sequence Detection System (Applied Biosystems, Foster City, CA) using a SYBR Green kit. The primer sequences used are listed in Table 1. The threshold was set in the linear range of fluorescence, and a threshold cycle (Ct) for each well was measured.

**Table 1:** Primers used for mRNA quantification by real-time RT-PCR.

Gene	Forward primer 5′-3′	Reverse primer 5′- 3′
mLDL-R	TGACTCAGACGAACAAGGCTG	ATCTAGGCAATCTCGGTCTCC
mHMG-CS	AACTGGTGCAGAAATCTCTAGC	GGTTGAATAGCTCAGAACTAGCC
mHMG-CR	CTTGTGGAATGCCTTGTGATTG	AGCCGAAGCAGCACATGAT
mFAS	GCTGCGGAAACTTCAGGAAAT	AGAGACGTGTCACTCCTGGACTT
mACS	GCTGCCGACGGGATCAG	TCCAGACACATTGAGCATGTCAT
mSCD-1	CCGGAGACCCCTTAGATCGA	TAGCCTGTAAAAGATTTCTGCAAACC
mβ-actin	GGCTGTATTCCCCTCCATCG	CCAGTTGGTAACAATGCCATGT
hLDL-R	AGCTGCCTCACAGAGGCTGA	CCAGAGCTTGGTGAGACATTG
hHMG-CS	TGTTTCAGATTGCAAGGTAATATGT	CCTGGCACCCAAAAGTTAAA
hHMG-CR	TACCATGTCAGGGGTACGTC	CAAGCCTAGAGACATAATCAT
hFAS	AACTCCAAGGACACAGTCACCAT	CAGCTGCTCCACGAACTCAA
hACS	AACCACGAACGCTTTGAGA	TCCAGATACATTGAGCATGTCAT
hSCD-1	TGTGTCCCAGATGCTGTCAT	CCCACCCAATCACAGAAAAG
hβ-actin	GGCATCCTCACCCTGAAGTA	AGGTGTGGTGCCAGATTTTC

The abbreviations are: LDL-R, low-density lipoprotein receptor; HMG-CR, 3-hydroxy-3-methylglutaryl coenzyme A reductase; HMG-CS, 3-hydroxy-3-methylglutaryl coenzyme A synthetase; FAS, fatty acid synthetase; ACS, acetyl-CoA synthetase; SCD-1, stearoyl-CoA desaturase 1. The prefix m indicates mouse and h human sequence.

### RNA interference

The shRNA expression vector pSH-ZsGreen-Neo, which expresses both Neomycin resistance gene and ZsGreen, was constructed by PCR amplification of Neomycin resistance gene (ORF) with SV40 early polyadenylation signal from pcDNA3.1(+) (Invitrogen) with primers 5′-ATTGGATCCGCCACCATGATTG AACAAGATGGATTGCACG-3′ and antisense 5′-ATTGGATCCGCTCGTATGTTG TGTGGAATTGTGA-3′ and inserted into BglII restriction site of pSIREN-Retro Q-ZsGreen under the control of CMV/MSV hybrid promoter. shNT and shORP8 were synthesized as oligonucleotides, annealed and inserted into pSH-ZsGreen-Neo via BamHI/EcoRI restriction sites. The oligonucleotides were: shNT sense, gatccGCATTGGTCGTCTCTATTAttcaagagaTAATAGAGACGACCAATGCttttttacgcg tG; antisense, aattcacgcgtaaaaaaGCATTGGTCGTCTCTATTAtctcttgaaT AATAGAG-ACGACCAATGCg; shORP8sense, gatccGAGTGGTCTTGCAAATTATttcaagaga-ATAATTTGCAAGACCACTCttttttg; antisense, aattcaaaaaaGAGTGGTCTTGCAAA-TTATtctcttgaaATAATTTGCAAGA CCACTCg. For generation of stably silenced cell lines, HuH7 cells were electrotransfected with pSH-ZsGreen-Neo constructs linearized using *Hin*dIII and selected by growth in 600 µg/ml G418 (Gibco) for 14 days. Single cell clones were generated by limited dilution and screened by Western blotting with anti-ORP8. Transient transfection of HuH7 cells with non-targeting or Nup62-specific siRNAs (sense GCAACAACCACACCUGCUAdTdT, antisense UAGCAGGUGUGGUUGUUGCdTdT) in combination with plasmid DNA was carried out using Lipofectamine 2000 (Invitrogen).

### Assay for cholesterol biosynthesis

HuH7 cells cultured on 6-well plates in EMEM (Sigma-Aldrich), 10% FBS, 20 mM HEPES, pH 7.4, without antibiotics, transfected with ORP8 cDNA or shRNA constructs, were washed with serum-free medium. Pulse-labeling was performed for 30 min at 37°C with [^3^H]acetic acid (500 µCi/well; GE Healthcare) followed by a chase period of 90 min in serum-free medium containing 25 mM mevalonate. After washing with PBS, cells were scraped into 900 µl of ice-cold 2% (w/v) NaCl. [^14^C]cholesterol was added to the cell lysates to correct the results for material losses. Aliquots of 100 µl were withdrawn for protein analysis. From the remaining 800 µl, lipids were extracted and separated by TLC on silica-gel plates by using petroleum ether/diethyl ether/acetic acid (60∶40∶1) as the solvent. The plates were dried and stained with iodine vapor. The cholesterol band was scraped, and [^3^H] and [^14^C] radioactivity was measured by liquid-scintillation counting. The data was normalized for cellular protein.

### Cholesterol binding assay

His_6_-ORP8 ORD was produced in *E.coli* Rosetta(DE3)™ (Novagen, Madison, WI), and purified on Ni-NTA agarose (Invitrogen) according to the manufacturer's instructions, but omitting detergent from the buffers. The major protein products in the purified preparation, with the apparent molecular masses of 72 and 67 kDa, were by mass-spectrometry analysis of tryptic peptides confirmed to represent the ORP8 ORD fusion at the Meilahti Medical Campus Protein Chemistry Unit.

His_6_-ORP8 ORD and GST as a negative control protein were used in an assay measuring the ability to extract [^3^H]cholesterol from unilamellar 400 nm diameter vesicles consisting of 99 mol% egg yolk phosphatidylcholine (Sigma-Aldrich), 1 mol% cholesterol (Sigma-Aldrich), and [^3^H]cholesterol (Life Technologies/Amersham TRL330, 44 Ci/mmol). The assays were performed with vesicle aliquots containing 100 pmol cholesterol, 9.9 nmol PC and 25–100 pmol purified protein according to [24].

### Yeast two-hybrid screening

Yeast two-hybrid screening was performed using Matchmaker™ GAL4 yeast two-hybrid system 3 (Clontech). cDNA encoding ORP8 lacking the 19 C-terminal amino acids (designated as ORP8m), which constitute a putative trans-membrane anchor, was amplified by PCR with the following primers. Sense: 5′-gatcattcatATGAGTCAGCGCCAAGGAAAAG; Antisense: 5′-AATGTCGACGTC TTTTTGTTGCAGAAAATAATCC. The amplified cDNA was cut with *Nde*I and *Sal*I and inserted pGBKT7 via the same restriction sites to construct bait plasmid pGBKT7-ORP8m. The bait plasmid was co-transformed with plasmids from Human Fetal Kidney MATCHMAKER cDNA Library (cat. no 638826, Clontech) into *Saccharomyces cerevisiae* AH109 strain according to the lithium acetate protocol as described in the manufacturer's manual. The plates were grown at +30°C in a humid incubator for one week, after which the colonies were subjected to x-gal filter assay for β-galactosidase activity. Plasmids in colonies positive for β-galactosidase activity were isolated from yeast and transformed into *E. coli* strain DH5α for sequencing.

### Bimolecular fluorescence complementation (BiFC) analysis

To construct BiFC plasmids, the sequences encoding fluorescent protein Venus residues 1–172 (Vn) or residues 154–238(Vc) were connected by C-terminus linker (GGGS)3 (for both Vn and Vc), or N-terminus linker RSIAT (for Vn) and RPACKIPNDLKQKVMNH (for Vc), respectively. Full-length *ORP8* cDNA (NM_001003712) and *Nup62* cDNA (NM_012346) were PCR amplified and inserted into BiFC vectors as fusions with the Vn and Vc fragments. Two combinations of fusion proteins were tested for BiFC efficency [25]. HuH7 cells were cotransformed with compatible plasmids encoding the fusion proteins ORP8/pVcN1 combined with Nup62/pVnC1, or ORP8/pVcC1 combined with Nup62/pVnC1, for identifing interaction of ORP8 and Nup62, or ORP4L/pVcN1 combined with Nup62/pVnC1 as a negative control. Plasmid pDsRed2-ER (Clontech) was cotransfected as a transfection efficiency control and as an ER marker. Overexpression of Nup62 is prone to result in cytosolic aggregates, as reported previously [26]. We therefore used a limited amount Nup62 plasmid and added 10 µg/ml cycloheximide to inhibit protein synthesis at 24 h after transfection, avoiding Nup62 cytosolic aggregates, and further incubated cells for 48 h to allow overexpressed Nup62 to mature. The cells were incubated for a total of 72 h at 37°C, and fluorescence emission was imaged at the RFP and GFP channels.

### Co-immunoprecipitation

Aliquots of 10^7^ Huh7 cells were washed twice with ice-cold PBS and incubated on ice 30 min with 1 ml lysis buffer (50 mM Tris–Cl, 150 mM NaCl, 0.5 mM MgCl_2_, 10% glycerol, and 0.5% Triton X-100, pH 8.0) with Protease Inhibitor Cocktail Set I (Calbiochem, San Diego, CA) and phosphatase inhibitor cocktails (Keygen Biotech, Nanjing, China). Cell lysates were centrifuged for 15 min at 15,000× g at 4°C. The supernatant was preabsorbed at 4°C for 1 h with 50 µl of rProtein G agarose (Invitrogen). The recovered supernatant was incubated with ORP8 or control antibody at 4°C overnight. The lysate–antibody mixture was added to fresh rProtein G agarose and incubated at 4°C on a roller for 3 h. Beads were washed five times with lysis buffer and boiled in 30 µl of 2× SDS-PAGE loading buffer. Samples were resolved on 12% SDS-polyacrylamide gels and subjected to Western blot analysis.

### Immunofluorescence microscopy

HuH7 cells transfected with *ORP8*/pcDNA4HisMaxC or the corresponding *ORP1L*, *ORP3*, or *ORP10* constructs using Lipofectamine 2000 (Invitrogen) for 24 h were fixed with 4% paraformaldehyde, permeabilized with 0.05% Triton X-100, and processed for indirect immunofluorescence microscopy using antibodies against the ORPs or Nup62 as previously described [27]. The specimens were analyzed with a TCS SP2 laser scanning confocal microscope (Leica, Wetzlar, Germany). Fluorescence intensity of nuclear circumference at the Nup62 (green) and ORP (red) channels was quantified by using the Leica LCS software.

## Results

### Adenoviral-mediated expression of ORP8 decreases plasma and hepatic lipids levels

**Figure 1:**
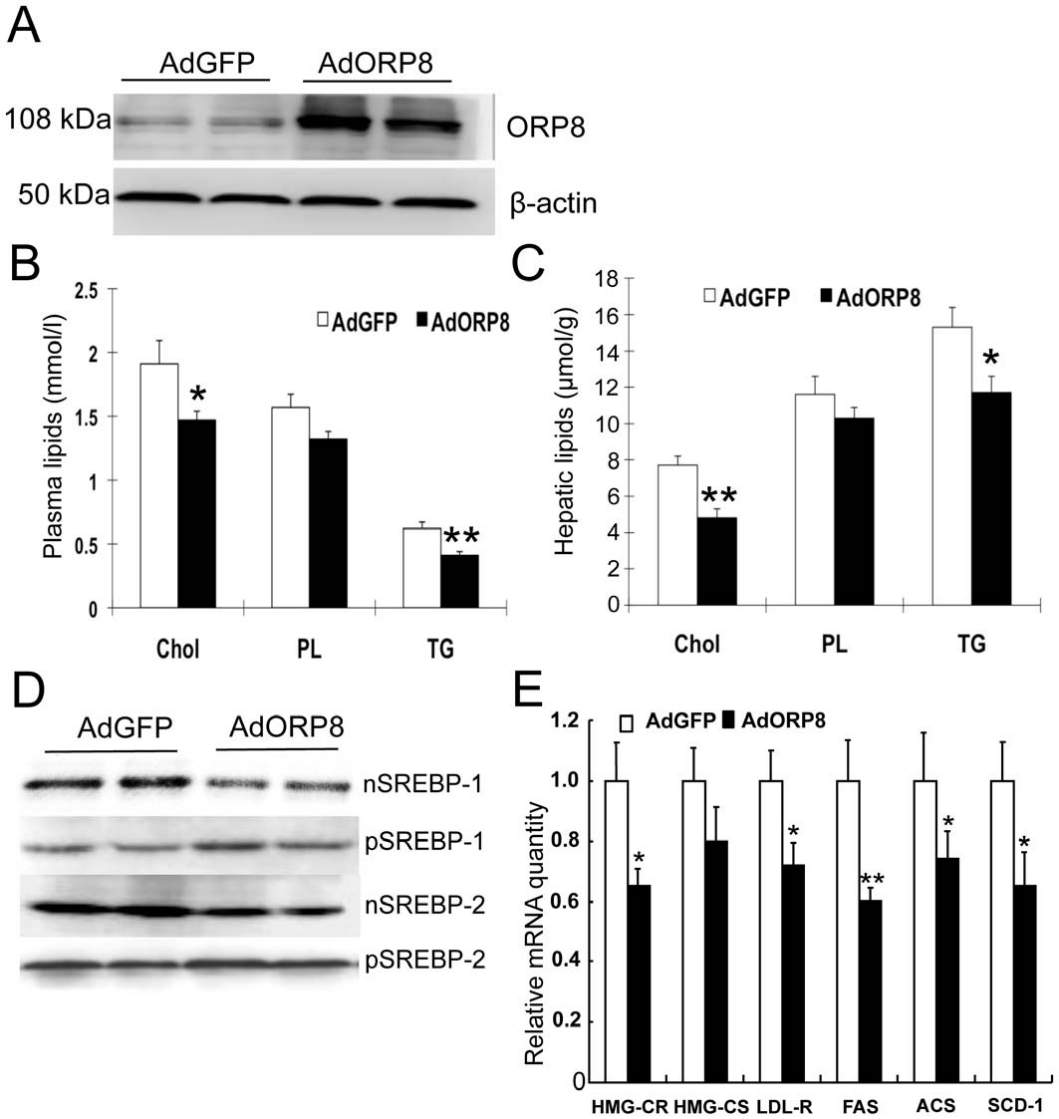
ORP8 overexpression reduces plasma and hepatic lipids in mice. **A** Western analysis of adenovirus (Ad)-mediated ORP8 overexpression in mouse liver. Liver total protein (20 µg/lane) from mice at day 5 after infection with AdGFP and AdORP8 was Western blotted with ORP8 antibody. **B** Plasma Cholesterol (Chol), choline phospholipids (PL), and triglycerides (TG) measured from animals at 5 days after infection with AdGFP or AdORP8. **C** Hepatic lipid levels of the mice at day 5. **D** Mouse liver nuclear (1st and 3rd panel) or total (2nd and 4th panel) protein fractions (40 µg/lane) at day 5 after infection were Western blotted with antibodies against SREBP-1 or SREBP-2. nSREBP, nuclear SREBP; pSREBP, precursor SBEBP. (E) Analysis of SREBP target gene mRNAs (identified at the bottom) in the liver of mice transduced with AdGFP (open bars) or AdORP8 (closed bars) by qPCR. The data represents mean ± s.e.m. (*p<0.05; **p<0.01; n = 5; t-test).

The recombinant adenovirus encoding ORP8 (AdORP8) or a control virus encoding GFP (AdGFP) was injected into female C57B/6 mice through the tail vein at the viral dose 3×10^8^ pfu per animal. This dose was found to result in a moderately elevated, 5-fold hepatic tissue ORP8 expression level as compared to animals injected with the control virus (Fig. 1A). The plasma total cholesterol, triglyceride (TG), and choline-containing phospholipid (PL) concentrations were determined for the mice at 5 days after AdORP8 injection and revealed a significant reduction of cholesterol (−34%) and TG (−37%), as well as tendency of reduced PL (−26%) as compared to AdGFP-transduced animals (Fig. 1B). Similarly, the liver tissue cholesterol (−40%) and TG (−24%) concentrations were markedly decreased in AdORP8-injected animals (Fig. 1C). In order to elucidate the underlying mechanism we analyzed by Western blotting the amount of SREBP-1 and -2 in nuclear fractions prepared from the liver of the transduced animals, as well as the precursor SREBPs (pSREBPs) in liver total protein preparations. This analysis revealed a significant reduction of both nuclear (n)SREBP-1 (−35%) and -2 (−38%) in the AdORP8-transduced animals, providing a plausible mechanistic explanation for the observed lipid changes. Coinciding with the reduction of nSREBPs, mild increases of the precursor forms of the SREBPs (pSREBP-1 and -2) were often observed (Fig. 1D). To validate the biological effect of the reduction of nSREBPs, we quantified the mRNAs for a number of SREBP-1 and SREBP-2 genes. This analysis revealed significant decreases of the *HMG-CR*, *LDL-R* (predominantly SREBP-2 target genes), *FAS*, *ACS*, and *SCD-1* (SREBP-1c target genes) mRNAs (Fig. 1E), consistent with the observed reduction of the nSREBPs.

#### ORP8 modulates SREBP-1 and -2 target gene expression and cholesterol biosynthesis in HuH7 cells

**Figure 2:**
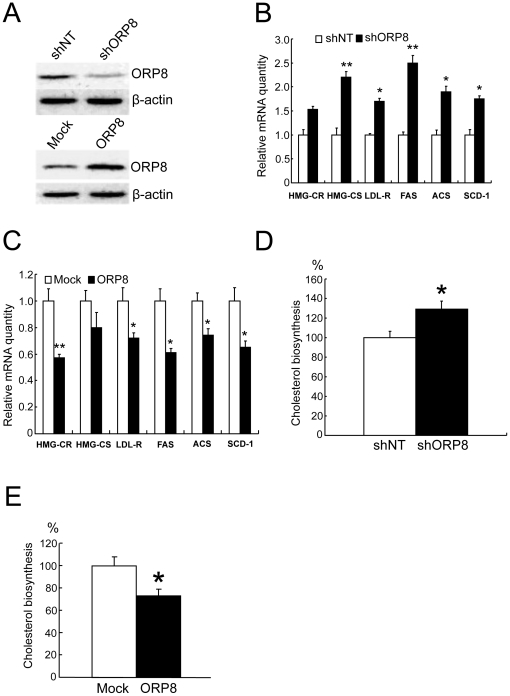
Silencing and overexpression of ORP8 have opposite effects on SREBP target gene expression in HuH7 cells. **A** Western analysis of HuH7 cells subjected to ORP8 silencing and overexpression. Stable expression of scrambled control shRNA (shNT) or shORP8, or overexpression by plasmid transfection for 36 h using Neon™ electroporation (ORP8; Mock = transfection with empty vector). **B** Quantification of selected SREBP target gene mRNAs (identified at the bottom) in cells expressing shNT or shORP8. **C** Quantification of selected mRNAs (identified at the bottom) in cells transfected with the empty vector (Mock) or *ORP8* cDNA (ORP8). **D** Cholesterol biosynthesis as measured by [^3^H]acetic acid incorporation (30 min pulse, 90 min chase), in cells expressing shNT or shORP8. The result is given as % of the biosynthesis in shNT-expressing cells. **E** Cholesterol biosynthesis in cells transfected with the plain vector (Mock) or *ORP8* cDNA (ORP8) for 36 h. The data represents mean ± s.e.m. (*p<0.05, **p<0.01, n = 4–6; t-test).

To further investigate ORP8 function in a hepatic cell model we employed the human hepatoma cell line HuH7 which is easily amenable to transfection. The cells were subjected to *ORP8* overexpression or silencing through RNA interference, followed by quantification of the mRNAs of the SREBP-2 target genes *HGM-CR*, *HMG-CS*, and *LDL-R*, and the SREBP-1 target genes *FAS*, *ACS*, and *SCD-1*. The *ORP8*-specific shRNA construct reduced the endogenous ORP8 expression by 65%, while the level of overexpression reached was 3-fold as compared to the endogenous level (Fig. 2A). ORP8 silencing induced moderate but significant increases of the *HMG-CS* (+110%) and *LDL-R* (+67%), *FAS* (+150%), *ACS* (+92%), and *SCD-1* (+75%) mRNAs (Fig. 2B), while ORP8 overexpression had the opposite effect, resulting in reduction of the *HMG-CR* (−41%), *LDL-R* (−28%), *FAS* (−40%), *ACS* (−25%), and *SCD-1* (−35%) mRNAs (Fig. 2C). To confirm that the mRNA changes observed are reflected in cellular lipid metabolism, we measured the incorporation of [^3^H]acetate into cholesterol during a 30-min pulse followed by 90-min chase, in HuH7 cells in which ORP8 was depleted or overexpressed. Cholesterol biosynthesis was moderately but significantly increased in HuH7 cells transfected with the ORP8 shRNA construct as compared to a non-targeting control (Fig. 2D), and decreased to a similar extent in cells overexpressing ORP8, as compared to cell transfected with the empty vector plasmid (Fig. 2E).

### ORP8 binds cholesterol

**Figure 3:**
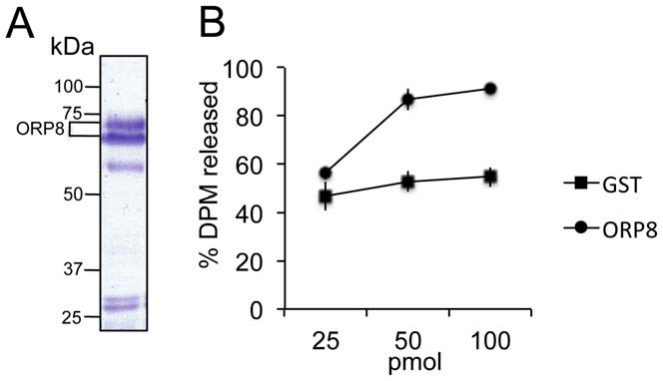
The ORP8 ligand-binding domain binds cholesterol. **A** The purified His_6_-ORP8 ORD preparation, Coomassie blue stained SDS-PAGE gel. The two major bands (indicated, ORP8) represent the ORP8 ORD fusion protein. **B** The ability of His_6_-ORP8 ORD to extract [^3^H]cholesterol from unilamellar PC∶cholesterol (99∶1 mol%) vesicles was assayed. Purified GST was used as a negative control. Each assay contained 9.9 nmol PC and 100 pmol cholesterol; The protein amounts used (25–100 pmol) are indicated at the bottom. The data represents % of total DPM extracted from the vesicles, a mean ± s.e.m. (GST, n = 4; His_6_-ORP8 ORD, n = 5).

To investigate whether ORP8 not only binds 25-hydroxycholesterol [19] but also cholesterol, we assayed the ability of purified His_6_-ORP8 ORD to extract [^3^H]cholesterol from unilamellar PC∶cholesterol (99∶1) vesicles. These experiments revealed dose-dependent extraction of cholesterol by His_6_-ORP8 ORD, which was significantly above the background release of [^3^H]cholesterol observed with the irrelevant control protein, GST (Fig. 3).

### ORP8 interacts physically with Nup62

To obtain further clues of ORP8 function we employed the yeast two-hybrid system to screen for interaction partners of ORP8m (missing the C-terminal membrane-spanning segment) employed as bait. A total of 1×10^6^ cotransformants were selected for quadruple prototrophy on SD/-Trp-Leu-His-Ade (SD/4-) plates, using a human embryonic kidney prey library. Of the 12 colonies growing under the selection and positive for β-galactosidase activity, eight represented cDNAs encoding nucleoporin of 62 kDa (Nup62, NM_153719).

**Figure 4:**
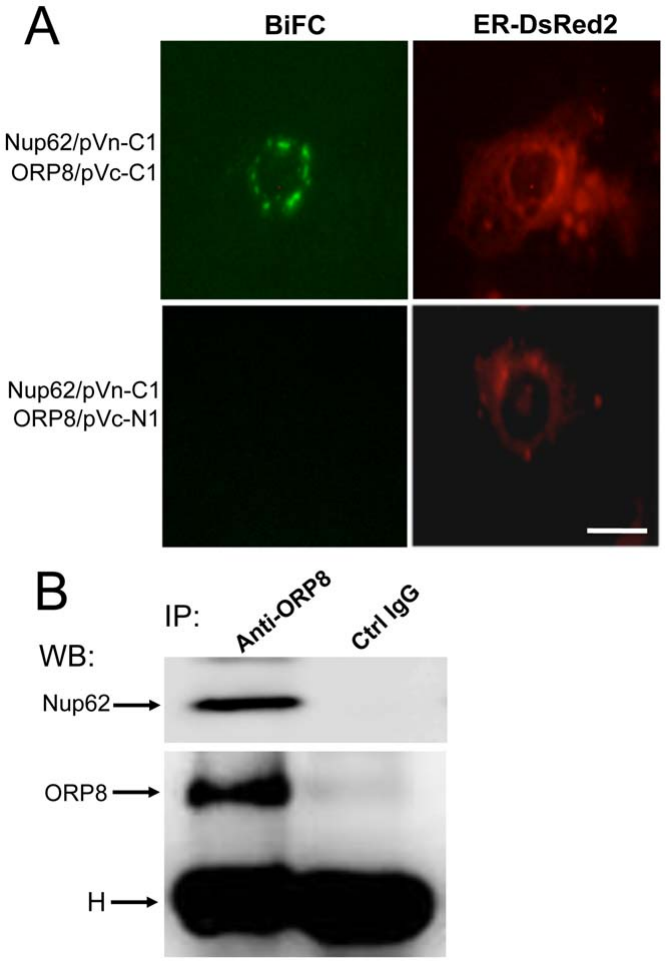
ORP8 interacts with Nup62. **A** Bimolecular fluorescence complementation (BiFC) analysis of ORP8 interaction with Nup62. HuH7 cells were cotransformed for 24 h with plasmids encoding the fusion proteins Nup62/pVn-C1 and ORP8/pVc-C1 or ORP8pVc-N1 (indicated on the left) for 24 h, followed by 48 h incubation with 10 µg/ml cycloheximide. ER-DsRed2 was contransfected as a transfection control and ER marker. BiFC (GFP channel) and DsRed fluorescence were imaged (identified at the top). Bar, 10 µm. **B** Lysate of untransfected HuH7 cells was immunoprecipitated with anti-ORP8 (identified at the top) or an irrelevant control IgG, followed by Western blot analysis with anti-Nup62 (top panel) or anti-ORP8 (bottom panel). H, IgG heavy chain.

To validate the result of the two-hybrid screen, we carried out bimolecular fluorescence complementation (BiFC) analysis and co-immunoprecipitation (co-IP) in HuH7 cells. Two BiFC combinations were tested under conditions in which further protein synthesis was inhibited with cycloheximide (CHX) after 24 h transfection: Nup62/pVn-C1 (Nup62 fused to the C-terminus of Vn fragment) with ORP8/pVc-C1 (ORP8 fused to the C-terminus of Vc fragment) showed a specific BiFC signal at pacthes at the nuclear circumference that co-localized with the ER marker ER-DsRed2, while Nup62/pVn-C1 with ORP8/pVc-N1 (ORP8 fused to the N-terminus of Vc fragment) showed no BiFC signal under the same conditions (Fig. 4A). The specific, spatially restricted signal given by the former fusion protein pair was markedly different from the extensive ER/nuclear envelope localization of ORP8 [19](see also later in this article). If CHX was not added, both combinations showed a BiFC signal in the cytoplasmic compartment apparently due to aggregation of the Nup62 fusion. No significant BiFC fluorescence was detectable in cells expressing ORP4L/pVc-N1 employed as a negative control and Nup62/pVn-C1 (data not shown). The BiFC analysis thus suggested a specific interaction of ORP8 with Nup62 at patchy structures at the nuclear circumference.

Formation of physical complexes between ORP8 and Nup62 was further confirmed by co-IP analysis, in which HuH7 cells were subjected to immunoprecipitation with anti-ORP8 or an irrelevant rabbit IgG. Western blot analysis of the immunoprecipitates revealed specific co-precipitation of Nup62 with ORP8 (Fig. 4B).

### Identification of the domains responsible for ORP8-Nup62 interaction

**Figure 5:**
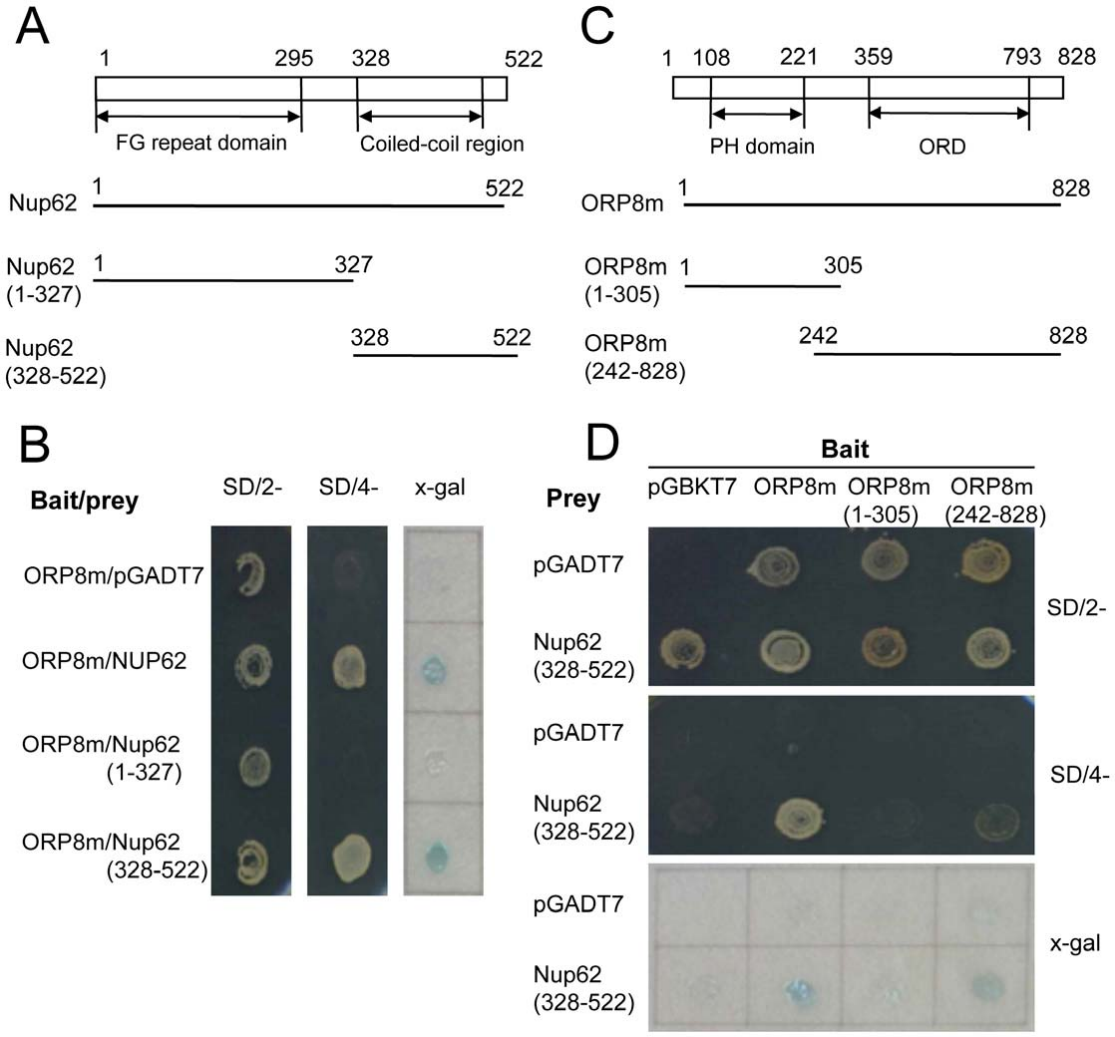
Identification of the interacting domains in Nup62 and ORP8. **A** Schematic presentation of the Nup62 prey constructs used in yeast two-hybrid assays. The numbers indicate amino acid positions. **B** Interaction of the Nup62 constructs and ORP8m (lacking the C-terminal trans-membrane segment). Yeast colonies grown on SD/2- plates (left), SD/4- plates (middle) and x-gal assay (right) are shown. **C** Schematic presentation of the ORP8 bait constructs used. **D** Interaction of the ORP8 constructs with Nup62 in the yeast two-hybrid assay. Yeast colonies grown on SD/2- plates (top), SD/4- plates (middle) and x-gal assay (bottom) are shown. pGADT7 is the empty prey vector and pGBKT7 the empty bait vector.

To identify which part of Nup62 interacts with ORP8, deletions of Nup62 were constructed in the prey vector pGADT7 (Fig. 5A). Their ability to interact with ORP8 was tested by two-hybrid assays. The Nup62 C-terminal fragment (aa 328–522) containing a coiled-coil forming region retained the interaction with ORP8m, whereas the N-terminal fragment (aa 1–327) with the FG repeat domain failed to support yeast growth on the selection medium or activity of the x-gal reporter (Fig. 5B), suggesting that the C-terminal part of Nup62 with a coiled-coil forming segment contains the determinant(s) for interaction with ORP8. To determine which region of ORP8 is involved in the interaction with Nup62, two deletions of ORP8 in bait vector pGBKT7 were made (Fig. 5C). The bait construct containing the C-terminal ORD of ORP8 (aa 242–834) grew on SD/4- selection plates and activated transcription of the x-gal reporter, whereas the N-terminal region containing the PH domain (aa 1–305), failed to interact with Nup62 (Fig. 5D). These results demonstrate that the ORP8 C-terminal region containing the ORD interacts with Nup62.

### Colocalization analysis of ORP8 and Nup62 by concocal microscopy

**Figure 6:**
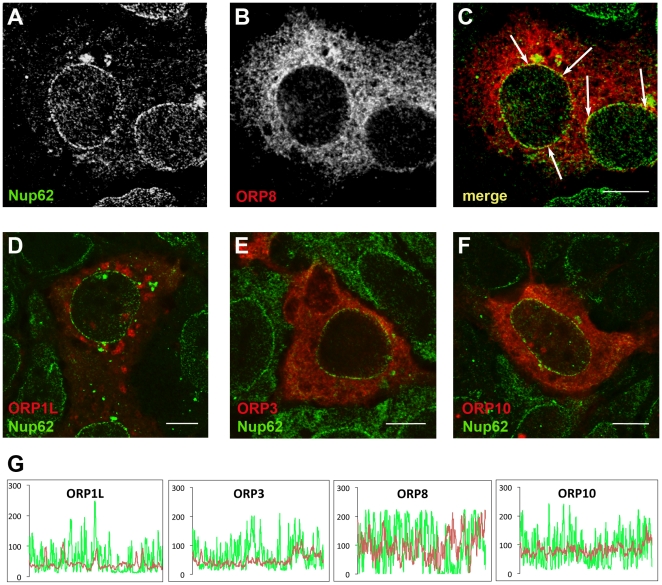
ORP8 co-localizes with Nup62 at the nuclear envelope: Confocal microscopy analysis. HuH7 cells were transfected with ORP8, ORP1L, ORP3, or ORP10 cDNA for 24 h using Lipofectamine 2000, followed by processing for confocal immunofluorescence microscopy double staining with anti-Nup62 (green) and anti-ORP (red) antibodies. **A–C** Nup62 and ORP8 localization in transfected Huh7 cells. Co-localization of ORP8 and Nup62 at the nuclear envelope is indicated with arrows in the channel merge panel. No Nup62 colocalization was observed with ORP1L (**D**), ORP3 (**E**), or ORP10 (**F**). Bars, 10 µm. **G** Analysis of ORP8, 1L, 3, or 10 (identified in the panels) colocalization with Nup62 at the nuclear envelope in representative cells. Fluorescence intensity (on an arbitrary scale) of the nuclear circumference at the Nup62 (green) and ORP (red) channels was quantified by using the Leica LCS software.

The subcellular localization of ORP8 in HuH7 cells was studied by immunofluorescence microscopy. Since the antibody available could not reliably detect the endogenous HuH7 protein, ORP8 was expressed in the cells by transient transfection, and cells were double stained with anti-ORP8 and anti-Nup62, followed by confocal fluorescence microscopy (Fig. 6A–C). ORP8 localized at reticular ER membranes and the nuclear envelope, at which significant co-localization with Nup62 was evident (arrows), consistent with results of the BiFC analysis (see Fig. 4A). For a comparison, we also analyzed the co-localization of Nup62 with three other ORPs, ORP1L, ORP3, and ORP10. ORP1L and ORP3 carry instead of a trans-membrane segment, a FFAT (two phenylalanines in an acidic tract) motif for ER targeting via the VAMP-associated proteins [28], while ORP10 lacks both the transmembrane segment and the FFAT motif. No significant co-localization of Nup62 with these ORP proteins was detected (Fig. 6D–E), consistent with a specific interaction of ORP8 with Nup62. Coincidence of the ORP and Nup62 stainings at the nuclear circumference was further analyzed by using the Leica LCS software, revealing marked colocalization of Nup62 with ORP8 and practically none with ORP1L and 3 (Fig. 6G). The ORP10 signal was somewhat above background levels at the nuclear envelope region, but did not coincide with the Nup62 fluorescence peaks.

### ORP8 interaction with Nup62 results in a decrease of nuclear SREBPs

**Figure 7:**
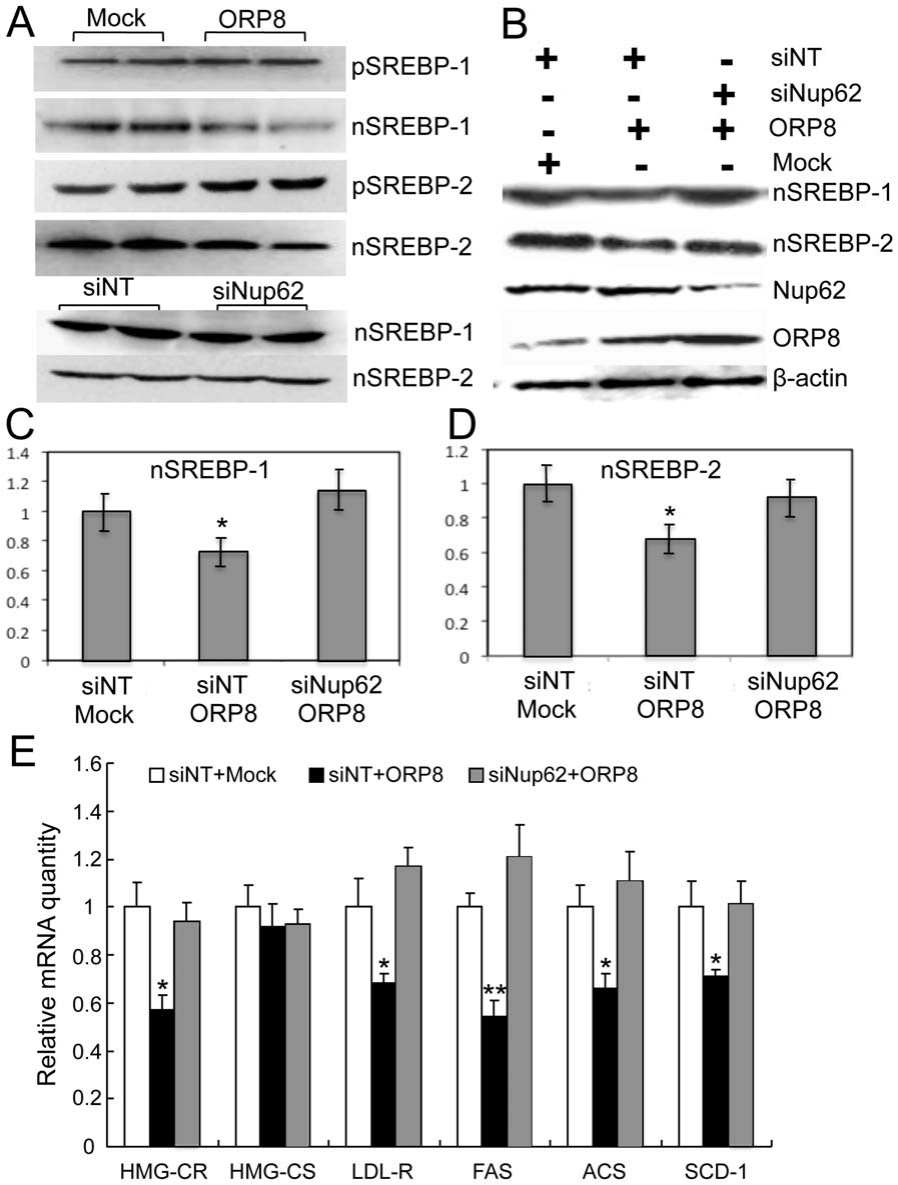
Nup62 is involved in reduction of nuclear SREBPs by excess ORP8. **A** HuH7 cells were transfected with empty vector plasmid (Mock), *ORP8* cDNA (ORP8), non-targeting siRNA (siNT) or siNup62 as indicated, followed by preparation of total protein specimens and nuclear fractions, and Western blot analysis thereof with antibodies against SREBP-1 and SREBP-2. Nuclear SREBPs (nSREBPs) decreased in cells overexpressing ORP8 (the top panels), while no change in nSREBPs was seen in cells transfected with siNup62 (the two bottom panels). **B** HuH7 cells were transfected with combinations of siRNAs and plasmids as indicated at the top. Nuclear fractions Western blotted for SREBP-1 and SREBP-2, and total protein specimens (40 µg/lane) blotted for Nup62, ORP8, and β-actin are shown at the bottom. **C,D** Quantification of the relative levels of nSREBP-1 and nSREBP-2 after the indicated combined transfections. **E** Effect of the combined transfections on the mRNA levels of SREBP target genes (identified at the bottom); qPCR analysis. The results represent mean ± s.e.m. (n = 4, *p<0.05, **p<0.01, t-test; difference to values of the siNT and Mock-transfected control, which was set at 1).

To investigate whether interaction of ORP8 with Nup62 could play a role in the effect of ORP8 on nuclear SREBPs, we transfected *ORP8* cDNA or *Nup62* siRNA (siNup62) into HuH7 cells. Like overexpression of ORP8 in liver tissue, excess of this protein reduced the amount of nSREBPs in HuH7 cells, while no change of nSREBPs was observed in HuH7 cells treated with *Nup62* siRNA (Fig. 7A). For further study, we designed experiments combining ORP8 overexpression and Nup62 silencing (Figure 7B–E). We transfected cells with empty vector plasmid (Mock) and non-targeting control siRNA (siNT), with *ORP8* cDNA and siNT, or with *ORP8* cDNA and siNup62, followed by analysis of nuclear SREBPs. Similar to the results in Fig. 7A, overexpression of ORP8 in the presence of siNT resulted in a moderate but significant decrease of nSREBP-1 and -2 as compared with Mock-transfected cells. Interestingly, this effect was reversed in cells subjected to knock-down of Nup62 (Fig. 7B–D), suggesting that a normal level of Nup62 is required for the reduction of nuclear SREBPs by excess ORP8. To validate the biological significance of the reversal phenotype induced in ORP8 overexpressing cells by Nup62 silencing, we quantified mRNAs for SREBP target genes in cells transfected with siNT+empty vector plasmid, siNT+ORP8 expression plasmid, or with siNup62+ORP8 expression plasmid. While ORP8 overexpression significantly dampened the expression of *HMG-CR*, *LDL-R*, *FAS*, *ACS*, and *SCD-1* mRNAs, silencing of Nup62 in the ORP8 expressing cells reversed the effect in all cases, bringing the mRNA levels back to control levels (Fig. 7E).

## Discussion

We previously identified OSBP-related protein 8 (ORP8) as an endoplasmic reticulum oxysterol receptor and implicated its role in cellular lipid metabolism [19]. In the present report, we demonstrate that moderate adenoviral overexpression of ORP8 in mouse liver significantly reduces the total cholesterol and triglyceride levels in both plasma and the liver tissue, concomitant with reduction of the nuclear forms of SREBP-1 and -2 and the mRNA levels of their target genes controlling cholesterol and fatty acid biosynthesis as well as LDL uptake. The results were replicated in the human hepatic cell line HuH7. Both overexpression and silencing of ORP8 in HuH7 cells had moderate but significant effects on the expression of SREBP-1 and -2 target genes. The observed reduction of nSREBPs provides a plausible mechanistic explanation for the observed effects on lipid homeostasis. We have earlier shown a related functional effect by another ORP family member, OSBP, whose overexpression was shown to increase the quantity of nSREBP-1 and the hepatic lipogenesis [29].

The reduction of plasma total cholesterol level induced by ORP8 coincided with a mild down-regulation of mRNAs encoding both enzymes of the cholesterol biosynthetic pathway and the LDL receptor. The experiments in the HuH7 cell model suggest that the reduction of plasma cholesterol may indeed be due to down-regulation of cholesterol biosynthesis. In mouse, the rate of hepatic cholesterol clearance is extremely high, resulting in markedly low serum LDL cholesterol levels [30]. Thus, even moderate down-regulation of LDL-R might lead to a quite significant elevation of LDL cholesterol. However, mouse is an HDL animal with approximately 80% of serum cholesterol found in the HDL fraction and, as shown by using tissue-specific inactivation of ATP-binding cassette transporter A1 (ABCA1), the liver produces at least 70% of serum HDL-cholesterol [31], [32]. It is therefore possible that reduction of cholesterol biosynthesis upon ORP8 overexpression may impact HDL cholesterol levels at an extent greater than the putative increase of LDL cholesterol due to LDL-R down-regulation.

Oxysterols are potent modulators of SREBP maturation. Radhakrishnan et al. [33] reported that oxysterols act by binding to Insigs, leading to an inhibition of the transport of Scap-SREBP complexes to the Golgi apparatus and proteolytic processing of the SREBP. We find it likely that ORP8 acts as a sterol sensor that responds to cellular concentrations of oxysterols or cholesterol, which we here show it binds, and, via a yet poorly understood mechanism, fine-tunes the activity of the SREBPs. Despite efforts, we have not been able to demonstrate effects of ORP8 manipulation on the suppression of SREBP processing or target gene expression upon exogenous oxysterol administration. However, we often detected a mild increase of pSREBPs in mouse liver or HuH7 cells overexpressing ORP8, indicating that ORP8 manipulation may, under normal growth conditions partially inhibit the proteolytic processing of the SREBP precursors. Interestingly, the present finding that ORP8 interacts with the nucleoporin Nup62 specifically at the nuclear envelope, as suggested by the BiFC analysis, provides a clue to a novel type of regulatory mechanism that may in part explain the impact of ORP8 on lipid homeostatic control. Experiments combining ORP8 overexpression with Nup62 knock-down suggested that depletion of Nup62 with siRNA can at least in part reverse the suppression of nSREBPs by ORP8. Reduction in the cellular level of Nup62 alone did not influence the amount of nSREBPs, suggesting that sequestering of Nup62 in complexes with ORP8 affects nSREBP quantity more strongly than a partial knock-down of Nup62 with siRNA. These findings are consistent with the hypothesis that a complex of ORP8 and Nup62 could modulate the nuclear transport of proteolytically activated SREBPs.

Steroid receptors and most of the nuclear factors involved in signaling cascades shuttle constantly between the nucleus and cytoplasm through the NPC [4]. Nup62 is present in two NPC modules, the Nup62-Nup58-Nup45-Nup54 and the Nup214-Nup88-Nup62 subcomplexes [34]. Nup62 is composed of two major domains: an intrinsically disordered FG-repeat containing N-terminal domain (aa 1–327) and an α-helical coiled-coil forming domain (aa 328–522). Nup62 is anchored at the NPC mainly via its coiled-coil domain [35]–[37]. Our data suggests that this domain also mediates binding to ORP8, possibly to a predicted coiled-coil forming segment located at amino acid residues 791–815 of ORP8, as determined with the COILS software (http://www.ch.embnet.org/software/COILS_form.html). The Nup62 FG-repeat domain, on the other hand, contributes directly to nucleocytoplasmic transport through interactions with nuclear transport receptors and with cargo [38], [39]. Nup62 interacts directly with importin-β [40] and with nuclear transport factor 2 (NTF2) through its FG-repeat domain [41]–[43]. In the case of NTF2, this interaction has been shown to be essential for its transport activity [38], [44]. Systematic analysis of nucleoporin dynamics predicts both transport and structural roles for Nup62 at the NPC [45], [46]. Furthermore, intermolecular sliding between components of the Nup62-Nup58-Nup45-Nup54 complex in the central channel of the NPC is thought to regulate pore diameter [36]. Nup62 mediates the nuclear import of the glucocorticoid receptor-hsp90 complex [47] and of the MUC1-C oncoprotein [48], and a more general role of Nup62 in protein traffic through NPC has been indicated [47]. SREBP-2 enters the nucleus through a direct interaction of its helix-loop-helix leucine zipper domain with importin-β [49], [50] However, the role of Nup62 in the transport of SREBP-2 or other SREBPs has remained unexplored. Our work indicates that ORP8 could, via interaction with Nup62, modulate the transport of SREBPs through the NPC. However, we want to emphasize that also other mechanisms are likely to contribute to the observed reduction on nSREBPs – a detailed mechanistic understanding of the functional role of the ORP8-Nup62 complex requires extensive future experimentation. A putative function of ORP8 in complex with Nup62 might also impact nuclear factors other than the SREBPs, and explain our earlier finding that ORP8 has the capacity to modify ATP-binding cassette transporter A1 (ABCA1) mRNA and protein expression in macrophage, the underlying mechanism of which remained largely unclear [19].

Like ORP8, its closest homologue in ORP subfamily IV, ORP5, is anchored to ER membranes via a C-terminal transmembrane segment and displays localization highly similar to that of ORP8, including distribution to the nuclear envelope [51]. Furthermore, Nup62 interacts with the sterol-binding domain of ORP8, a region highly conserved between ORP5 and ORP8 [16]. It is therefore possible that also ORP5 could interact with Nup62, although we at this point have no evidence for such an interaction.

The present findings suggest that ORP8 has the capacity to regulate hepatic lipogenesis and plasma lipid levels, apparently through modulation of the quantity of the active nuclear forms of SREBPs. Although the underlying mechanisms remain incompletely understood, we find it likely that the newly discovered interaction of ORP8 with Nup62 may be involved. This finding also adds a novel, intriguing aspect in the study of OSBP-related protein function. Future work with an ORP8 knock-out mouse model under construction is necessary for a comprehensive understanding of the role that this protein plays in lipid metabolism.
